# Influence of Primary Light Exposure on the Morphophysiological Characteristics and Phenolic Compounds Accumulation of a Tea Callus Culture (*Camellia sinensis* L.)

**DOI:** 10.3390/ijms251910420

**Published:** 2024-09-27

**Authors:** Maria Y. Zubova, Evgenia A. Goncharuk, Tatiana L. Nechaeva, Maria A. Aksenova, Georgiy P. Zaitsev, Vera M. Katanskaya, Varvara V. Kazantseva, Natalia V. Zagoskina

**Affiliations:** 1 K.A. Timiryazev Institute of Plant Physiology, Russian Academy of Sciences, 127276 Moscow, Russia; goncharuk.ewgenia@yandex.ru (E.A.G.); nechaevatatyana.07@yandex.ru (T.L.N.); akse.masha@yandex.ru (M.A.A.); vera@katanski.com (V.M.K.); k.v.-90@mail.ru (V.V.K.); 2 All-Russia National Research Institute of Viticulture and Winemaking “Magarach”, Russian Academy of Sciences, 298600 Yalta, Russia; gorg-83@mail.ru

**Keywords:** tea, in vitro cultures, morphology, phenolic compounds, phenylpropanoids, catechins, stress

## Abstract

Tea plant calli (*Camellia sinensis* L.) are characterized by the accumulation of various phenolic compounds (PC)—substances with high antioxidant activity. However, there is still no clarity on the response of tea cells to light exposure of varying intensity. The purpose of the research was to study tea callus cultures grown under the influence of primary exposure to different light intensities (50, 75, and 100 µmol·m^−2^·s^−1^). The cultures’ growth, morphology, content of malondialdehyde and photosynthetic pigments (chlorophyll *a* and *b*), accumulation of various PC, including phenylpropanoids and flavanols, and the composition of catechins were analyzed. Primary exposure to different light intensities led to the formation of chloroplasts in tea calli, which was more pronounced at 100 µmol·m^−2^·s^−1^. Significant similarity in the growth dynamics of cultures, accumulation of pigments, and content of malondialdehyde and various phenolics in tea calli grown at light intensities of 50 and 75 µmol·m^−2^·s^−1^ has been established, which is not typical for calli grown at 100 µmol·m^−2^·s^−1^. According to data collected using high-performance liquid chromatography, (+)-catechin, (−)-epicatechin, epigallocatechin, gallocatechin gallate, epicatechin gallate, and epigallocatechin gallate were the main components of the tea callus culture’s phenolic complex. Its content changed under the influence of primary exposure to light, reaching the greatest accumulation in the final stages of growth, and depended on the light intensity. The data obtained indicate changes in the morphophysiological and biochemical characteristics of tea callus cultures, including the accumulation of PC and their individual representatives under primary exposure to light exposure of varying intensity, which is most pronounced at its highest values (100 µmol·m^−2^·s^−1^).

## 1. Introduction

Light is one of the abiotic factors necessary for the growth and development of plants. Its role is especially important in the early stages, during the transition from etiolated growth to deetiolation, that is, photomorphogenesis [1]. It is during this period that chloroplasts form in cells, serving as sites for realization of photosynthesis—a process that supplies energy sources (ATP) and reducing agents (NADPH_2_) for various metabolic processes [2]. Light is important for seed germination, stem and leaf formation, phototropism, the opening/closing of stomata, flowering induction, the regulation of various metabolic processes, and more [3,4,5,6,7].

The plants’ vital activity and productivity depend on the intensity and duration of light exposure. A change in the light intensity led to changes in their morphophysiological characteristics and influenced an increase in the biomass of aboveground organs and the amount of photosynthetic pigments, hormones, and other metabolites [2,8,9]. Plant ontogenesis, in particular, the transition to flowering and tuberization, depended on the duration and photoperiod of light exposure [5,10,11].

It should also be noted that, like any abiotic factors, light under certain conditions can also have a stressful effect on plants. This effect is due to the accumulation of reactive oxygen species (ROS) in cells and the development of oxidative stress [12,13]. The decrease in the potentially destructive effect of ROS is due to the functional activity of the antioxidant system and the presence of the excessive accumulation of various antioxidants in plant cells, including polyphenols (PC) [3,14].

PC are one of the most common secondary metabolites and are synthesized in almost all plant cells [15]. They are represented mainly by monomeric substances, such as hydroxybenzoic acids, phenylpropanoids, and flavonoids [6]. In addition, PC oligomers are also formed in plants. These include proanthocyanidins and lignans, which are derivatives of flavanols (catechins) and phenylpropanoids (coniferyl alcohol), respectively [15]. The phenolic polymer lignin should not be forgotten, as it is an important component of plant cell walls [16].

Currently, significant progress has been made in the study of PC biosynthesis, which is carried out with the participation of the following two pathways: shikimate and acetate-malonate (polyketide) [17]. Almost all stages are known; many genes, transcription factors and enzymes responsible for the transformation of PC have been identified [6,15,17,18,19].

The important role of PC in regulating the growth of plants and their interaction with the environment is known. They are important components of the processes of photosynthesis and respiration, auxin transport, interaction with pathogens, stress resistance, and adaptation [15,20]. The latter case is due to the functional role of PC as one of the components of the antioxidant cell protection system capable of inactivating ROS and preventing their toxic effect [21]. The antioxidant activity of these representatives of secondary metabolism is determined by their structure—the number of aromatic rings and double bonds and the number and location of functional groups [22].

It has been reported that the antioxidant activity of some plant PC exceeds that of ascorbic acid and tocopherol; it also persists even when these compounds enter the human body with food [23]. There is evidence that the consumption of flavonoids in effective amounts reduces the risk of pathogenesis caused by oxidative stress and chronic inflammatory processes [24]. PC are promising in the treatment of diseases associated with obesity, as well as in the fight against infectious and viral diseases, including COVID-19 [15,25]. The significant role of PC for the prevention of cardiovascular diseases, atherosclerosis, diabetes mellitus, and cancer has been noted [25,26]. This confirms the need to study these representatives of secondary metabolism and search for producing plants, as well as factors regulating their formation. In this case, the in vitro culture of plant tissues can also be considered the main platform for these purposes [27,28].

The unquestioned advantages of plant cell cultures are growing in controlled conditions; the absence of restrictions in the action of seasonal, climatic, and geographical factors; the preservation of the ability to form metabolites characteristic of intact plants; and the possibility of modulating their biosynthesis using different exogenous impacts [27,29]. It should also be noted that the full potential of callus plant culture technology has not yet been exploited; the time has come to develop and market more callus culture-based products [30].

When working with in vitro cell and tissue cultures, the choice of an object, the direction of its metabolism, and the value of the products obtained from it for industrial or pharmaceutical use are of major concern [31,32]. In this case, the tea plant (*Camellia sinensis* L., family Theaceae), an important horticultural crop growing in tropical and subtropical latitudes, is a subject of great interest [26].

The tea plant metabolome is represented by many compounds of both primary and secondary metabolism, which include PC [26,33,34,35,36]. The main components of the phenolic tea complex are flavan-3-ols, or catechins, which account for up to 75% of the total PC content in the leaves. They are represented by simple and gallated compounds, such as (+)-catechin, (−)-epicatechin, (−)-gallocatechin, (−)-gallocatechin gallate, (−)-epigallocatechin, (−)-epicatechin gallate, and (−)-epigallocatechin gallate [33,34]. Tea plants are also characterized by the formation of simple PC, which are represented by gallic, *p*-coumaric, and caffeic acids, as well as their derivatives—theogallin and chlorogenic acid [36]. The PC of tea have P-vitamin capillary-strengthening activity and have a positive effect on the treatment of human diseases, as well as reducing the risk of other undesirable effects of stress on the body [37,38].

In recent years, considerable interest has been paid to the in vitro study of tea plant cultures as a potential producer of biologically active substances of a phenolic nature [39,40,41]. These cultures retained the ability to accumulate PC, including such flavanols as (+)-catechin and (−)-epicatechin, which are characteristic of the original explants [42]. An increase in the biosynthetic capacity of tea cultures in vitro was achieved by the action of various exogenous factors, including light [1,4]. It was reported that the total accumulation if PC increased during the long-term cultivation of tea calli at a light intensity of 3600 lux [43]. However, the response of tea plant cells to primary exposure to light of varying intensities, which can be considered a regulator of their metabolic activity, has not yet been studied, including the accumulation of phenolic antioxidants in in vitro cultures.

Our goal was to study the response of tea callus cultures to primary exposure to light of varying intensities during the cultivation cycle. To achieve this, we evaluated the morphology of calli, as well as the growth activity, the content of malondialdehyde as an indicator of the presence of a stress response, the total accumulation of PC, including phenylpropanoids and flavonols, and the balance of individual components of the catechol complex using high-performance liquid chromatography. The analysis of the various PC class content makes it possible to assess the regulation of the biogenesis of these plant metabolites in in vitro tea cultures under the influence of an abiotic factor such as light.

## 2. Results

### 2.1. The Effects of Light on Tea Callus Culture Morphophysiological Parameters

**Figure 1:**
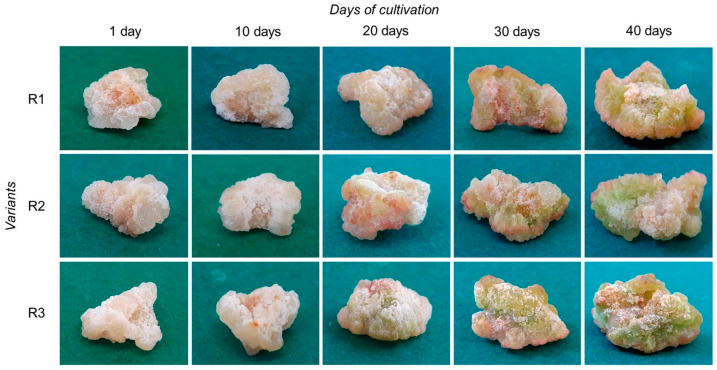
Tea callus cultures grown under the following different intensities of light exposure during the cultivation cycle (40 days): R1—50 µmol·m^−2^·s^−1^; R2—75 µmol·m^−2^·s^−1^; R3—100 µmol·m^−2^·s^−1^.

The morphology of tea callus cultures has changed over time (Figure 1). At the beginning of the light exposure (the first to tenth days), the calli of all variants were compact, quite dense, and light beige in color. The formation of new cells (white) on the surface of the calli was noted, which is more pronounced in the R1 and R2 variants (Figure 1 and Figure S1). On the 20th day, the formation of cells with a pink color was noted on the callus surface in most variants. This pigmentation in variant R1 was not so vividly expressed.

For variant R3, the presence of light green pigmentation was shown, which indicated the formation of chlorophyll-containing cells. On the 30th day, this process was characteristic of all calli variants. It was most pronounced in variant R3. By the 40th day, all calli acquired a green color of varying intensity, as follows: variant R1—light green, variant R2—green, variant R3—bright green. It should also be noted that during the entire cycle of cultivation, in light of varying intensity, the calli had compact structures and were dense.

**Figure 2:**
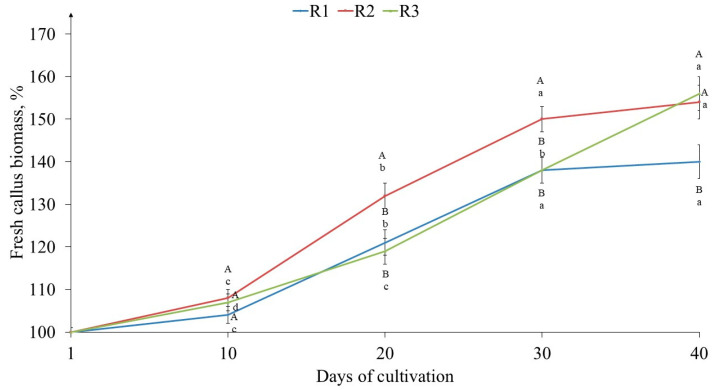
Biomass growth dynamics of tea callus cultures grown under the following different intensities of light exposure during the cultivation cycle (40 days): R1—50 µmol·m^−2^·s^−1^; R2—75 µmol·m^−2^·s^−1^; R3—100 µmol·m^−2^·s^−1^. Results are expressed as means ± SDs, *n* = 3. The significant differences at *p* < 0.05 are denoted by different Latin letters, as follows: uppercase letters indicate significant differences between different light intensities in alphabetic order from highest to lowest, and lowercase letters indicate significant differences between duration of light exposure in alphabetic order from highest to lowest. Pairwise multiple comparisons were carried out using Tukey’s range test.

The callus biomass accumulation of tea culture was recorded for 40 days, at 10-day intervals, under various light intensities. According to the data obtained, the R1 and R2 variants are characterized by a typical S-shaped growth curve (Figure 2). The durations of their lag, linear, and stationary phases were equal. The increase in biomass in variant R1 at almost all stages of growth was lower relative to variant R2, and by the 40th day, the biomass increased in R1 and R2 were 140% and 154%, respectively. In calli grown at the highest light intensity (variant R3), the durations of the lag and linear growth phases were similar to those of variant R1. However, after the 30th day, its growth continued, and by the 40th day, it reached 156%, which was similar to variant R2.

The water content in tea calli of all variants had a maximum value on day 1 of light exposure (Table 1). This indicator significantly and equally (by 2.5%) decreased by day 10. With further light exposure, the water content in the tea calli of each variant changed slightly.

Chlorophyll *a* and *b* content in tea callus cultures grown under different light intensities was estimated using fluorescence intensity (FI) (Table 2).

At the initial stages of growth (10th day), the FI of chlorophyll *a* in tea calli of all variants was very low, especially in the R3 variant. On the 20th day, this parameter increased by 3.2, 4, and 4.5 times in the R1, R2, and R3 variants, respectively. By the 30th day of light exposure, the chlorophyll *a* FI significantly exceeded that of 20 days in the R1 and R2 variants by 4 and 5.5 times, respectively, while in the R3 variant, it exceeded the 20th day FI by 1.2 times. As a result, in R1 и R3 variants’ FI of chlorophyll *a* became statistically equal and exceeded that of variant R2 by 18%. By the 40th day of light exposure, the FI of chlorophyll *a* in variants R1 and R2 was equal and exceeded that of the 30th day by 1.5 times. In the R3 variant, these changes were more pronounced (by 2.3 times), and R3 reached its maximum value relative to other variants of light intensity.

In all variants of tea callus cultures grown at different light intensities, the FI of chlorophyll *b* was always significantly lower than that of chlorophyll *a* (Table 2). On the 10th day of the light exposure, the FI of chlorophyll *b* was the smallest and closest to the values of R2 and R3. On the 20th day of light exposure, this indicator increased relative to the 10th day by 2.8, 3, and 23 times in the R1, R2, and R3 variants, respectively. The FI of chlorophyll *b* on the 30th day under light exposure significantly increased only in the R1 and R2 variants (by 3.6 and 4.5 times, respectively). As a result of these changes, equal FI values of chlorophyll *b* were observed in all studied variants. By the 40th day, the FI of chlorophyll *b* doubled in all variants compared to the 30th day, reaching its maximum value. It was statistically equal for the R1 and R2 variants and higher (by 20%) for the R3 variant.

All this indicates changes in the nature of the growth, pigmentation, and FI of chlorophyll *a* and *b* in vitro under primary exposure to light of varying intensity.

### 2.2. The Effects of Light on Tea Callus Culture Lipid Peroxidation Level

**Figure 3:**
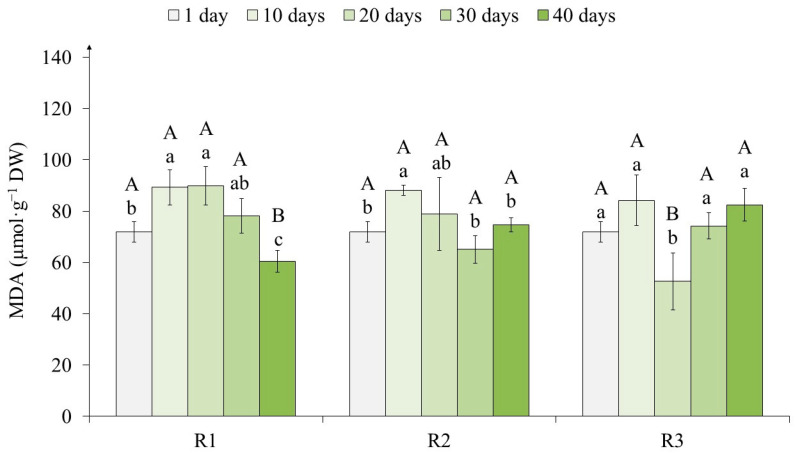
The malondialdehyde (MDA) content in tea callus cultures grown under the following different intensities of light exposure during the cultivation cycle (40 days): R1—50 µmol·m^−2^·s^−1^; R2—75 µmol·m^−2^·s^−1^; R3—100 µmol·m^−2^·s^−1^. Results are expressed as means ± SDs, *n* = 3. The significant differences at *p* < 0.05 are denoted by different Latin letters, as follows: uppercase letters indicate significant differences between different light intensities in alphabetic order from highest to lowest, and lowercase letters indicate significant differences between duration of light exposure in alphabetic order from highest to lowest. Pairwise multiple comparisons were carried out using Tukey’s range test.

The MDA content in the tea callus cultures changed as they grew under different light intensities (Figure 3). First of all, it should be noted that from the first to the 10th day of primary exposure to light, the MDA content increased by 16% in all variants.

On the 20th day of light exposure, the MDA content in variants R1 and R2 remained at the same level, whereas in variant R3, it decreased by 40% relative to the 10th day. On the 30th day, a similar trend was observed for R1 and R2, in contrast to R3, in which this indicator increased (by 20%). With further light exposure (40 days), the MDA content decreased in R1 (by 22%) and did not change in R2 and R3.

This indicates a change in the level of LPO in tea callus cultures exposed to primary light of different intensities, which was noted to a greater extent in the R1 variant.

### 2.3. The Effects of Light on Tea Callus Culture Phenolic Content

**Figure 4:**
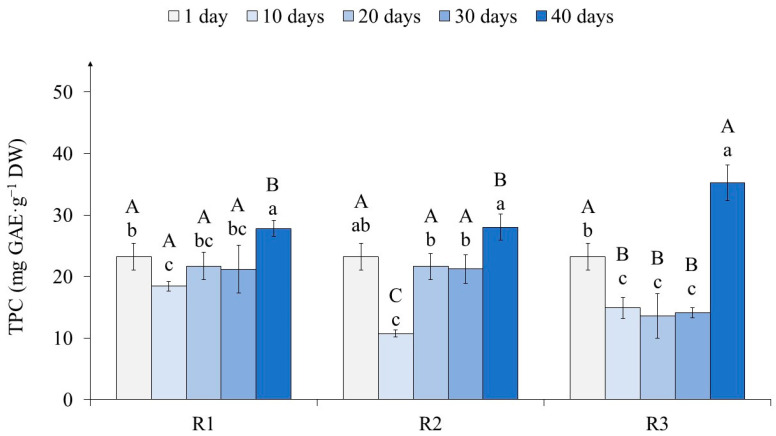
The total phenolic content (TPC) in tea callus cultures grown under the following different intensities of light exposure during the cultivation cycle (40 days): R1—50 µmol·m^−2^·s^−1^; R2—75 µmol·m^−2^·s^−1^; R3—100 µmol·m^−2^·s^−1^. Results are expressed as means ± SDs, *n* = 3. The significant differences at *p* < 0.05 are denoted by different Latin letters, as follows: uppercase letters indicate significant differences between different light intensities in alphabetic order from highest to lowest, and lowercase letters indicate significant differences between duration of light exposure in alphabetic order from highest to lowest. Pairwise multiple comparisons were carried out using Tukey’s range test.

Determination of the total phenolic content (TPC) showed that from the first to the tenth day of light exposure, its level decreased, especially in R2 (2.2 times) (Figure 4).

On the 20th day of light exposure, the amount of TPC did not change in variants R1 and R3 and increased in R2 by almost two times. On the 30th day, in each variant, this indicator remained at the same level. By the 40th day, in the tea calli of variants R1 and R2, TPC increased equally relative to the 30th day (by 25%), while in variant R3, it increased by 60%, which contributed to the greatest accumulation of PC. Moreover, in the final stages of culture growth (40 days), the amounts of these secondary metabolites were at their maximum in all variants, as follows: equal in R1 and R2 and 30% higher in R3.

**Figure 5:**
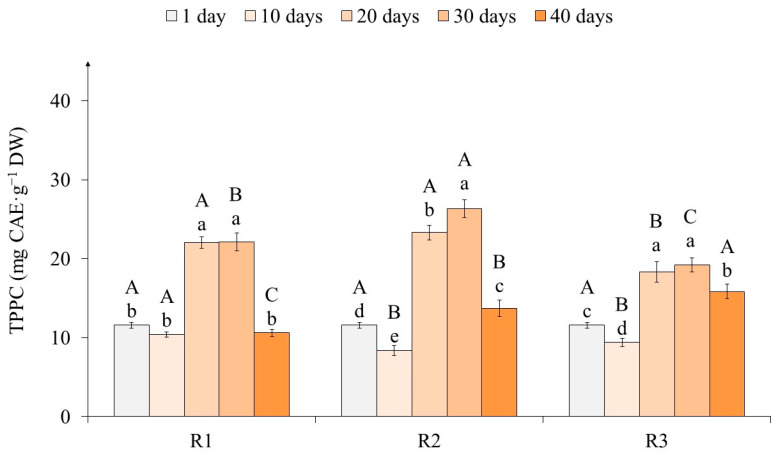
The total phenylpropanoid content (TPPC) in tea callus cultures grown under the following different intensities of light exposure during the cultivation cycle (40 days): R1—50 µmol·m^−2^·s^−1^; R2—75 µmol·m^−2^·s^−1^; R3—100 µmol·m^−2^·s^−1^. Results are expressed as means ± SDs, *n* = 3. The significant differences at *p* < 0.05 are denoted by different Latin letters, as follows: uppercase letters indicate significant differences between different light intensities in alphabetic order from highest to lowest, and lowercase letters indicate significant differences between duration of light exposure in alphabetic order from highest to lowest. Pairwise multiple comparisons were carried out using Tukey’s range test.

The determination of the total phenylpropanoid content (TPPC) did not reveal significant changes in their level at the beginning of the primary exposure to light of different intensities on tea callus cultures (Figure 5). From the first to the tenth day of light exposure, the TPPC decreased in R1, R2, and R3 by 10, 27, and 19% respectively.

By the 20th day of light exposure, the TPPC in tea callus cultures increased significantly (in the R1 and R3 variants by 50% and in variant R2 by 60%, relative to the 10th day) and remained at this level until the 30th day. By the 40th day, this indicator decreased as follows: in variants R1 and R2 by almost twice and in variant R3 by 17%.

This indicates more pronounced changes in the TPPC content in tea callus cultures of variants R1 and R2 compared with variant R3.

**Figure 6:**
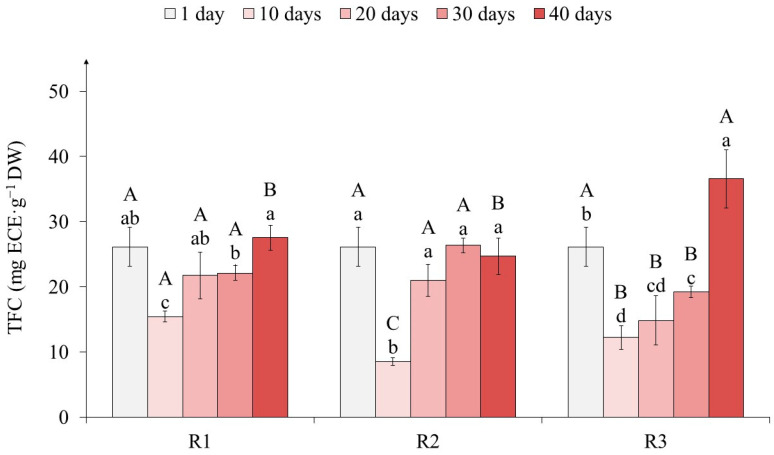
The total flavanol content (TFC) in tea callus cultures grown under the following different intensities of light exposure during the cultivation cycle (40 days): R1—50 µmol·m^−2^·s^−1^; R2—75 µmol·m^−2^·s^−1^; R3—100 µmol·m^−2^·s^−1^. Results are expressed as means ± SDs, *n* = 3. The significant differences at *p* < 0.05 are denoted by different Latin letters, as follows: uppercase letters indicate significant differences between different light intensities in alphabetic order from highest to lowest, and lowercase letters indicate significant differences between duration of light exposure in alphabetic order from highest to lowest. Pairwise multiple comparisons were carried out using Tukey’s range test.

Determination of the total flavanol content (TFC) showed a significant decrease in tea callus cultures from the first to the tenth day of primary exposure to light of different intensities (Figure 6). Thus, for variants R1 and R3, it decreased by 40% and 55%, respectively, while for variant R2, it decreased by 70%.

By the 20th day, the TFC increased only in variants R1 and R2 relative to the 10th day—by 1.5 and 2.5 times, respectively—and remained at this level until the 30th day. A different tendency was observed on the 40th day of light exposure, when the accumulation of these metabolites increased in the R1 and R3 variants (by 1.2 and 2 times, respectively) relative to the 30th day, but did not change in the R2 variant. It should also be noted that at the end of the tea callus culture growth under different light intensities, the TFC in the R1 and R2 variants was almost equal, while in variant R3, it was higher (by 30%).

### 2.4. The Effects of Light on Tea Callus Culture Catechin Complex

Determination of the individual components of the catechin complex in tea callus cultures using high-performance liquid chromatography (HPLC) showed significant similarity in their composition but differences in the individual component contents (Table 3 and Table S2). First of all, it should be noted that in all the studied variants of tea callus cultures, the accumulation of (−)-epicatechin (EC) was the greatest. High levels were also noted for (+)-catechin (C), epicatechin gallate (ECG), and gallocatechin gallate (GCG). The content of epigallocatechin (EGC), especially epigallocatechin gallate (EGCG), was significantly lower.

It should also be noted that the contents of individual components of the catechin complex in tea callus cultures reached higher values in the final stages of exposure to light of varying intensity, as follows: in R1 and R3 on the 40th day and in R2 on the 30th day. From the first to the tenth day of light exposure, there was an insignificant decrease (17–27%) in all variants, except for EGCG, the amount of which did not change in R1 and increased in R3 (by 40%). By the 20th day of light exposure, the changes in the accumulation of individual catechins were of a different nature. In the R1 variant, only the EC content increased slightly (by 15%), while the amounts of other components of the catechin complex decreased relative to the 10th day, especially EGC and GCG (by 40%). In the R2 variant, during this period, not only the EC content but also the C and GCG contents increased (by 54, 60, and 65%, respectively); the other components of the catechin complex remained at the same level. A different trend is typical for R3, in which the number of all individual components of the catechin complex decreased (from 50 to 60% relative to the 10th day of light exposure). On the 30th day of light exposure, there were no changes in the content of most components of the catechin complex in the R1 variant, except for an increase in C and EGC levels (by 70% and 60%, respectively) relative to the 20th day of light exposure. In the R2 variant, the accumulation of these metabolites in most cases increased in the following series: EGC by 180%, ECG by 126%, EC by 86%, GCG by 60%, and C by 53%. The exception was EGCG, the content of which did not change. As for the R3 variant, in this case, the greatest increase was noted for C (by 295%) and to a lesser extent for GCG and ECG (by 139% and 125%, respectively), as well as EGC and ECG (by 88% and 55%, respectively). On the 40th day of the light exposure, the contents of most compounds of the catechin complex in the variants R1 and R3 increased relative to the 30th day, while in the R2 variant, it decreased. The R1 variant had an equal and significant increase in EC and GCG (by 240%) content and to a lesser extent, ECG (by 155%), as well as EGC (by 130%), and the amount of EGCG did not change. In R2, the contents of all components of the catechin complex were 18–25% lower than those of the 30th day of light exposure, with the exception of EGCG, the level of which did not change. In R3, the amounts of metabolites of a catechin nature, such as EC (by 180%), GCG (by 75%), and C and EGC (by 45%) increased. As for EGC and EGCG, their contents decreased (by 32 and 50%, respectively).

This indicates a difference in the accumulation of various compounds of the catechin complex in tea callus cultures grown at different intensities of light.

## 3. Discussion

The important role of light in the regulation of morphophysiological and biochemical characteristics of plants is known [1,8]. It is an essential factor for the formation of chloroplasts—sources of energy and metabolites necessary for the functional activity of cells [9,11]. Its effect leads to the activation of photorespiration, the opening/closing of stomata, the regulation of flowering processes, and plant productivity, including the formation of pharmacologically valuable metabolites [3]. Despite the rather long history of studying the light regulatory role in relation to many physiological and biochemical processes in plant organs, tissues, and cells, there is still an opinion about the specificity of their response (especially during primary exposure) and the dependence on the intensity of light’s action [7,14]. In addition, in this case, great opportunities are provided by in vitro plant cultures, which are grown under strictly controlled conditions, consist of cells with a low level of differentiation, and retain many of the properties of the original explants [30,44]. In addition, due to the increasing risk of environmental impacts and the need to preserve plant raw materials, including industrial ones, understanding the mechanisms and methods of regulating the production capacity of cell cultures, including when using exposure to light, is important for the biotechnological production of valuable plant metabolites. This is also applied to PC, which are substances with high antioxidant activity that are successfully used in the treatment of human diseases of various etiologies [23,24].

### 3.1. The Effects of Light on Tea Callus Culture Morphophysiological Parameters

The assessment of the morphophysiological parameters of in vitro plant cultures under the influence of various factors is one of the important stages of research [45]. This is of particular interest in relation to the primary effect of different light intensities, which corresponds to the transition from scotomorphogenesis (growth in the dark) to photomorphogenesis (growth in the light) [2] at a simpler level of structural organization compared to that of an intact plant.

One of the features of in vitro plant cultures is the heterogeneity of calli, manifested in their shape, size, and level of differentiation [46]. To a certain extent, this is also typical for tea calli grown at different light exposure intensities (Figure 1). At the lowest light intensity (50 µmol·m^−^^2^·s^−^^1^), which we tested earlier [47] and, to a certain extent, served as a control over the effect of higher doses (75 and 100 µmol·m^−2^·s^−1^), changes in the morphology of cultures at all stages of the cultivation cycle were less pronounced relative to other variants.

The formation of white cells occurs on the surface of callus cultures during the cultivation cycle, which is associated with the activity of meristematic zones [46,48]. The same process is typical for callus cultures of the tea plant grown under the conditions of primary exposure to light of varying intensity (Figure 1 and Figure S1). The formation of these cells was more pronounced under lower light intensities (variants R1 and especially R2) and during the linear growth phase (up to 30 days of light exposure). In addition, there were single areas with pink-red cells on the surface of the tea calli of all variants starting from the 20th day of the light exposure. We assume, based on the literature data on tea plants, that the pinkish-red pigmentation of single cells of tea callus cultures is due to the accumulation of anthocyanins, as is typical for the leaves of young shoots of an intact plant [49]. However, this assumption requires further study.

Tea callus cultures exposed to primary light of varying intensities are also characterized by the formation of cells with green pigmentation (Figure 1). This is due to the formation of photosynthetic pigments in chloroplasts, which is characteristic of the initial stages of chloroplastogenesis in plants [9,50]. Green pigmentation was present in all the variants but had different degrees of manifestation, as follows: R1 < R2 < R3. This is consistent with the data put forth by other researchers, which indicate that the activation of chloroplastogenesis in callus cultures depends on the intensity and duration of light exposure [7,9,51].

Once again, it confirms the regulatory role of light on the processes of cell differentiation and cells’ functional activity, as is typical for the initial stages of plant photomorphogenesis [8]. Moreover, in tea cultures grown at high light intensity (variant R3), all these changes manifested themselves at earlier stages of growth, and they were more pronounced relative to the cultures grown at lower light intensities (R1 and R2 variants).

Thus, the primary exposure to light of different intensities led to changes in the tea callus cultures’ morphologies during the growing cycle. This was a consequence of alterations in their intertissue and intracellular differentiation, in particular due to the formation of chloroplasts—important suppliers of energy and metabolites for the vital activity of plant cells [3]. All these transformations can affect the biomass increase in tea calli grown under different light intensities.

It should be noted that tea callus cultures are characterized by a low growth rate, which was noted not only by us [42] but also by other authors [52]. When it was grown under the conditions of different light intensities, the increase in biomass in all the variants at the end of the passage did not exceed 156%, which was typical for the R2 and R3 variants (Figure 2). In the R1 variant, which was grown at low light intensity, this indicator was lower. Based on these data, it can be concluded that tea callus growth is better at higher light intensities (75 μmol·m^−2^·s^−1^ and 100 μmol·m^−2^·s^−1^). Differences in the growth dynamics of tea plant callus cultures grown under different light intensities were also noted over time. Thus, at its lower values (R1 and R2 variants), a transition to the stationary growth phase was noted (from 30 to 40 days of cultivation), which is not typical for its highest value (variant R3), in which the linear growth phase continued. Perhaps this effect is a specific reaction of tea cells grown at high light intensities, which is characterized by the greatest greening of the callus surface. The relationship between chlorophyll accumulation and plant growth has been noted by a number of authors [53,54].

The water content in plant tissues is an important indicator when assessing their viability and resistance to exogenous influences [55]. In tea calli grown at different intensities of light exposure, this indicator had similar and rather high values in all variants during the growing cycle (Table 1 and Table S1). According to the literature data, this evidences their low mitotic activity [56] and absence of significant changes in cell elongation [57].

All of the above indicates the possibility of growth regulation in tea calli exposed to light of different intensities. The largest increase in biomass was observed at light intensities of 75 and 100 μmol·m^−2^·s^−1^. The greatest changes in growth dynamics were noted at high values, as was noted in the leaf-derived callus of *Solanum xanthocarpum* cultivated under the influence of white light with an intensity of 45–50 μmol·m^−2^·s^−1^ [58]. For *Crataegus aronia* calli, a more intensive increase in biomass was also observed at higher lighting intensities (60 and 150 µmol m^−2^·s^−1^) [59]. The in vitro cultivation of plant cell cultures under conditions of light exposure is accompanied by the formation of chloroplasts, and this process depends on the light’s intensity [57,59]. The relationship between chlorophyll accumulation and plant growth has been noted by a number of authors [53,54].

The formation of photosynthetic pigments, such as chlorophylls *a* and *b*, is one of the important morphophysiological parameters of plant tissues [9]. They are responsible for absorbing light energy and its further conversion into the energy of chemical bonds, which is necessary for many metabolic processes in plants [59].

The formation of chlorophylls *a* and *b* is also typical for tea calli (Table 2). The FI of chlorophyll *b* was always significantly lower than that of chlorophyll *a*, as is typical for plants [7]. At the same time, in the first days (from 10 to 20) of the light exposure, there was a significant increase in the total FI level of these photosynthetic pigments of tea callus grown at the highest intensity of light exposure (variant R3) relative to its lower values (variants R1 and R2). With further light exposure, no such differences were noted between the variants.

Thus, the formation of photosynthetic pigments in tea callus cultures depends on the intensity and duration of light exposure, which is confirmed by the statistical analysis data (Table S1). In all cases, it reaches its maximum level by the end of the passage, which was more evident under the action of high light intensity (variant R3). This is consistent with the ideas about the important role of photosynthetic pigments in light absorption and the photoprotection of cells from high exposure [51,60]. As for the functional activity of photosynthesis in in vitro plant cultures grown in light conditions, this is a debatable issue [9,57]. Differences in the structure of chloroplasts formed in callus tissues from those of intact plant cells, as well as in their photosynthetic activity, have been reported [61].

In conclusion, it should be noted that the primary effect of different light intensities on tea callus cultures led to changes in their morphology. This was manifested in the formation of cells with different pigmentation on their surface—pink-red and green (with varying degrees of saturation). As for the formation of chlorophyll, this is an integral attribute of the differentiation of plant cells under conditions of light exposure, as was observed in in vitro tea cultures. In addition, in this case, a direct relationship was clearly observed between the intensity of light exposure and the accumulation of photosynthetic pigments. The important role of light signaling, including its intensity and duration, in regulation has been reported in the literature [60,62]. At the same time, there were no significant changes in the growth dynamics of tea calli grown at different intensities of light exposure. In all the studied variants, the increase in biomass by the end of the passage had almost equal values, as well as water content. The only exception was the tea culture grown at high light intensity (variant R3), which was characterized by the highest content of photosynthetic pigments and the absence of a transition to the stationary growth phase on the 40th day of cultivation relative to the other variants. All this indicates that the primary exposure to light of varying intensity affected the growth, morphology, and formation of photosynthetic pigments in tea callus cultures. All these changes were more pronounced at high values of light flux (100 µmol·m^−2^·s^−1^) and long exposure.

### 3.2. The Effects of Light on Tea Callus Culture Peroxidation Level

Lipid peroxidation (LPO) is an important indicator in assessing the physiological state of plants and their response to the influence of various exogenous factors [20]. Its increase due to an imbalance between the formation and utilization of ROS indicates the presence of a stress response in plants [63]. This also applies to light, which under certain conditions, including during primary exposure, can act as a stressor [64,65].

The determination of MDA content as an indicator of LPO showed its almost equal increase in all tea callus cultures at the initial stage of the action of exposure to light of varying intensity, namely, from 1 to 10 days (Figure 3). This indicated, firstly, the presence of a stress response in the tea callus cultures, as is typical for the primary response of cells to the action of an exogenous factor [13]. Secondly, this effect indicated a nonspecific reaction of tea cultures to exposure to light of different intensities, since this parameter was equal in all the variants of treatment.

Other trends were observed as tea cultures continued to grow under different light intensities. In variants R1 and R2, the MDA content decreased by the end of the light exposure. This indicates its adaptation to light exposure, which is probably due to the activation of antioxidant defense systems, including high- and low-molecular weight antioxidants [20]. In the R3 variant, this indicator has hardly changed, which indicates a more pronounced stress response of tea cultures grown under conditions of high light intensity. The presence of a stress response in plant cells in in vivo in vitro conditions, when light flux values are changing, was also reported by other authors [1,11,64,65].

All of the above indicates that the primary exposure to light had a stressful effect on in vitro tea cultures, and this effect did not depend on the light’s intensity (Table S1). The increase in the LPO levels in the cultures was equal in the first days of exposure to light of varying intensity. Its decrease was noted in the final stages of their growth, which is more typical for tea calli grown at low light intensity (variants R1 and R3). This indicates the adaptation of in vitro cultures to exposure to light and the formation of ROS defense systems arising under these conditions. To a certain extent, this may be a consequence of the accumulation of PC in them, which are effective bioantioxidants [20,24,25]. The only exception was the tea culture grown under high light intensity (R3), in which the LPO level was generally high throughout the passage. The presence of a stress response in plant cells at high light flux values was also reported by other authors [1,11].

### 3.3. The Effects of Light on Tea Callus Culture Phenolic Content

PC are among the most common secondary metabolites in plants, the formation of which is characteristic of almost all cells [27]. Their antioxidant activity and participation in plant protection from various environmental factors, including light, are known [28]. On the one hand, it is important for their life activity, and on the other hand, it can act as a stressor, increasing the formation of ROS [12]. In this case, PC play an important role in their inactivation [66].

To clarify the response of tea callus cultures to the primary exposure to light of various intensities, we used spectrophotometric methods for determining various classes of PC. They are successfully used by many researchers to assess both their total accumulation and the balance of various classes [42,66,67].

The initial stages of light exposure (1–10 days) were accompanied by a decrease in the amount of TPC in tea callus cultures of all variants (Figure 4). A more pronounced effect was observed under the action of high light intensities (variants R2 and R3) relative to its lower values (variant R1). This is consistent with the data of other researchers on a decrease in the accumulation of PC in plant tissues under the primary action of light exposure [68].

With a longer light exposure (up to 30 days), there were two tendencies in tea callus TPC change, as follows: the stability of this indicator, which was typical for R1 and R3, and its increase, which is typical for R2. At the same time, the lowest TRS value is typical for calli grown at high light exposure (100 µmol·m^−2^·s^−1^). This indicates a significant decrease in their ability to biosynthesize PC relative to crops grown at lower light exposure intensities and characterized by an almost equal accumulation of these secondary metabolites. It should also be noted that on the 40th day of light exposure, TPC in callus cultures of all variants (especially in R3) increased dramatically, reaching its maximum value.

All of the above indicates the regulatory role of light on the accumulation of TPC in tea callus cultures, which depends on both the intensity and duration of exposure (Table S1). The fact that light intensity, duration of exposure, and spectral composition lead to significant changes in the accumulation of PC both in plants and in vitro cultures has been reported by many authors [7,9,15]. To a large extent, this may be due to the formation of chloroplasts as one of the effective centers for the formation of polyphenols [14,18]. We cannot exclude the presence of a stress reaction during the primary exposure of plant cells to light, which in turn is accompanied by changes in the antioxidant defense system, including the accumulation of phenolic bioantioxidants [7,13].

Phenylpropanoids are the biogenetically earliest phenolic metabolites, which are formed at the initial stages of phenolic metabolism [15]. They can not only accumulate in plant cells but also serve as precursors in the biosynthesis of various flavonoids [17].

The determination of TPPC using the spectrophotometric method makes it possible to primarily assess their balance in plant tissues, which relieves and accelerates their initial assessment according to this indicator [59]. The TPPC accumulation had similar trends in tea callus cultures grown at different light intensities (Figure 5). Its manifestation was more pronounced in R1 and R2 relative to R3. In all variants, the lowest TPPC values are noted at the initial (1–10 days) and final (40 days) stages of light exposure, and the maximum values are noted in the intermediate period (20–30 days). All these changes are close to the circadian rhythm of biological processes and reflect the nature of these phenolic metabolites’ accumulation. At the same time, under conditions of high light exposure (100 µmol·m^−2^·s^−1^), the changes are expressed to a much lesser extent relative to its lower values (50 and 75 µmol·m^−2^·s^−1^).

The statistical processing of the data revealed a significant effect of light intensity and duration on tea callus cultures (Table S1). This trend in the formation of these phenolic metabolites is likely due not only to their role as antioxidants but also to their role as precursors in the biosynthesis of flavonoids, which include the flavanols discussed in this article [17].

Flavanols are representatives of the largest class of PC, namely flavonoids [32,38]. They are the main components of the tea plant phenolic complex, as well as the callus cultures initiated from the tea plant [33,47]. It should be noted that the TFC in the leaves of young shoots is 70%, while in callus cultures, it does not exceed 60% [41].

According to the data obtained, the nature of the response of tea callus cultures to the effect of varying light intensity at the TFC level is largely close to that of TFC (Figure 6). Perhaps this is a consequence of their dominance in the phenolic complex of in vitro cultures. At the same time, it should be noted that there is a lower accumulation of the TFC value relative to the initial (1 day) and final (40 days) stages of light exposure, which is more typical for R3. It is important to emphasize that the maximum TFC was observed only in the callus culture of tea at the final stage of the high-intensity light action (100 µmol·m^−2^·s^−1^).

Consequently, the TFC in tea callus cultures depends not only on the light intensity at which they are grown but also on the duration of its exposure (Table S1). We cannot exclude the relationship between the TFC and photosynthetic pigment accumulation. The latter to a certain extent indicates the process of cell differentiation and the formation of chloroplasts, as well as one of the sites of PC biosynthesis [9,50].

Our studies demonstrate differences in the accumulation of various classes of PC, in particular TPPC and TFC, in callus cultures of the tea plant grown under primary exposure to light of varying intensity. This was manifested in a decrease in TPC, and especially in TFC, at the primary stages of light exposure (1–10 days), which is not typical for TPPC.

This is probably associated with the catabolism of PC, including flavanols, under changed conditions, which depended on the light intensity and was more pronounced at high doses. The fact that PC can be degraded and serve as energy sources and substrates for the formation of other compounds has been reported in the literature [69]. To a certain extent, the result of these changes was an increase in TPPC (20–30 days), demonstrating the activation of the initial stages of phenolic metabolism and the accumulation of phenylpropanoids. The accumulation of these biogenetically early phenolic metabolites under the action of light has been reported in the literature [16,70]. However, at the final stage of light exposure (40 days), the TPPC decreased in all variants, which may be a consequence of their role as precursors in the biosynthesis of other classes of PC [17,70,71]. This is consistent with our data on the higher content of TPC in callus cultures of this period of light exposure and minor changes in TFC. All this is of considerable interest for further study of the regulation of various stages of phenolic metabolism in tea callus cultures under the action of light exposure, as well as the study of genes and transcription factors involved in this process.

### 3.4. The Effects of Light on the Tea Callus Culture Catechin Complex

One of the unique features of tea plants and the callus cultures obtained from them is the ability to form flavanols, which include flavonoid compounds such as flavan-3-ols or catechins [15,38].

The spectrum of these compounds is diverse and is represented by C, EC, EGC, and gallocatechin (GC) and their respective gallate esters—EGCG, ECG, GCG, and CG [72,73]. They are characterized by high antioxidant activity, exceeding that of well-known antioxidants such as ascorbic acid, tocopherol, and trolox [38,73]. The consequence of this is significant interest in tea plant catechins, which are successfully used as pharmacologically valuable plant substances for the health of the population.

**Figure 7:**
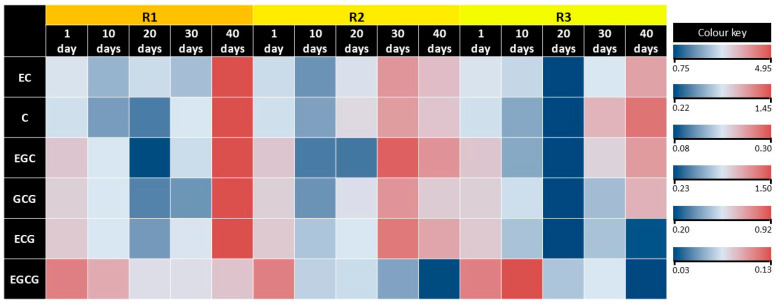
Heatmap showing the individual components content of catechins in tea callus cultures grown under the following different intensities of light exposure during the cultivation cycle (40 days): R1—50 µmol·m^−2^·s^−1^; R2—75 µmol·m^−2^·s^−1^; R3—100 µmol·m^−2^·s^−1^. EC—(−)-epicatechin; C—(+)-catechin; ECG—epicatechin gallate; GCG—gallocatechin gallate; EGC—epigallocatechin; EGCG—epigallocatechin gallate.

According to HPLC data, catechins of tea callus cultures grown under light of different intensities were represented by their simple (C, EC, EGC) and gallated (GCG, ECG, EGCG) forms (Table 3, Figure 7 and Figure S2). The main compound catechin complex of all the variants was EC. Whereasthe contents of C, GCG, and ECG were lower (by three times on average). The lowest accumulation is typical for gallated forms of flavan-3-ols, such as EGC and EGCG. This may be a consequence of the peculiarities of their formation at the final stages of flavonoid biosynthesis [34,49,74,75].

According to our data, the contents of all components of the catechin complex in callus tea cultures decreased in the first days of light exposure (1–10 days), which is consistent with TFC data. This indicates the suppression of the biosynthesis of these compounds of the flavonoid pathway [74]. It should also be noted that the greatest accumulation of certain compounds of catechin nature (EC, C, EGC, GCG, sometimes ECG) in tea callus cultures was noted only with prolonged exposure to light of varying intensity, as follows: in R1 and R3, on day 40 and in R2, earlier, on day 30. The statistical processing of the data revealed a significant effect of light intensity and duration (Table S1).

It should be noted that the largest quantity of these metabolites occurred under the action of low light intensity (R1). Based on this finding, it can be concluded that at high values of this indicator, such pronounced activation of catechin formation did not occur, which may depend on the expression of catechin-related genes (ANR, ANS, LAR, C4H, PAL, CHI, CHS, and DFR), as noted in the work by Zhang et al. [36]. One fact about EGCG is of interest. This is a unique metabolite of tea plants, which has received much attention from researchers due to its high antioxidant activity and wide spectrum of pharmacological action [4]. However, in tea callus cultures, its accumulation is minimal relative to all other catechins. However, in R3, its rapid accumulation was noted in the first days of light exposure (day 10), which is not typical for all other components of the catechin complex. This effect of light exposure is very interesting and deserves further research due to the pharmacological value of this metabolite.

In conclusion, it should be noted that the greatest accumulation of catechins, represented by both simple and gallated forms, was noted at the final stages of growth of tea callus cultures grown at low light intensity, that is, timed to its stationary growth phase. To a certain extent, this effect was also noted for cultures grown at higher doses of light, although it was less pronounced. Based on this finding, we can conclude that light has a regulatory role in the primary effect of light on the biosynthesis of catechins.

## 4. Materials and Methods

### 4.1. Plant Material and General Growth Conditions

The object of this study was callus cultures grown in vitro, obtained from the stem of young shoots of a tea plant (*Camellia sinensis* L., Georgian variety). For their growth, Heller’s nutrient medium [76] containing 2,4-dichlorophenoxyacetic acid (5 mg L^−1^), glucose (25 g L^−1^), and agar [Bacto Agar Typ USA, Ferak, Berlin, Germany] (7 g L^−1^) was used [77]. Calli were grown in the climatic chamber at +26 °C, with relative air humidity of 70%, in the dark. The subcultivation duration was 40 days.

**Figure 8:**
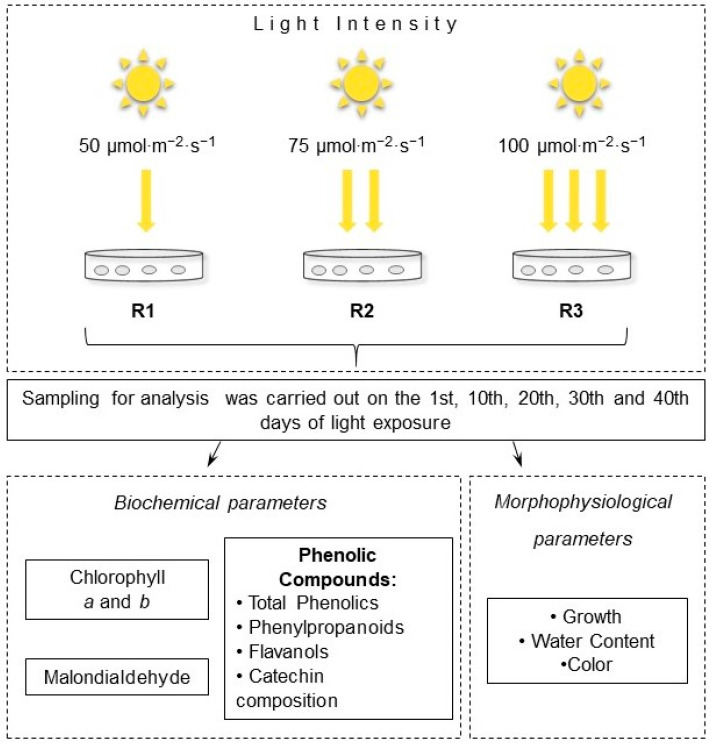
The design of the experiment and analyzed parameters.

During the experiment, tea calli were placed in sterile Petri dishes (Medpolymer, Russia) containing 30 mL of growth Heller’s nutrient medium with the same composition. Six calli were placed in each cup, and the fresh weight (FW) of each one was 150–180 mg. Cultures were grown in the climatic chamber in conditions of a 16/8 h light/dark regimen and different light intensities, as follows: 50 µmol·m^−2^·s^−1^, 75 µmol·m^−2^·s^−1^, and 100 µmol·m^−2^·s^−1^ (Philips TL–D 58 W/33-640 fluorescent lamps, Poland), which corresponded to the R1, R2, and R3 variants (Figure 8). The spectra of the lamps (Figure S3) were recorded using an LI-180 spectrometer (LI-COR, Lincoln, NE, USA). The light intensity was controlled by an LI-190R quantum sensor (LI-COR, Lincoln, NE, USA).

In experimental tea calli, starting from day 1 and every 10 days for 4 weeks (the 1st, 10th, 20th, 30th, and 40th days of culture growth), the morphological and physiological parameters were measured. Some cultures were fixed in liquid nitrogen and stored at −70 °C until other biochemical analyses were performed.

### 4.2. Determination of Morphological Parameters of Tea Callus Cultures

The morphology of tea callus cultures was assessed according to the following criteria: color, density, and the presence of pigments [78]. Lateral sections of calli were viewed on a SteReo LumarV12 stereoscopic microscope (Carl Zeiss, Jena, Germany). Photos were taken using a Nikon COOLPIX P600 camera (Tokyo, Japan).

### 4.3. Determination of Tea Callus Culture Growth

The growth of callus tissue was assessed using the change in fresh weight over the study period and expressed as a percentage [79]. To achieve this, the callus weight was determined at the beginning (W_i_) and the end (W_f_) of subcultivation (sampling periods for analysis), according to the following formula: (W_f_/W_i_) × 100%.

### 4.4. Determination of the Water Content in Tea Callus Cultures

To determine the water content, the calli (150 mg FW each) were dried (BD-115 thermostat, Binder, Tuttlingen, Germany) to a constant weight (+70 °C, 48 h). The water content was determined using the difference between the FW and dry weight (DW) and expressed as a percentage of the water content in the callus [80].

### 4.5. Determination of the Chlorophyll a and b Fluorescence Intensity in Tea Callus Cultures

To determine the chlorophyll content, tea calli (100 mg FW each) were extracted with 96% ethanol [81]. Chlorophylls *a* and *b* were determined using an RF-5301PC spectrofluorophotometer (Shimadzu, Tokyo, Japan) at the following corresponding excitation and emission wavelengths: for chlorophyll *a*, λex = 440 nm and λem = 600−800 nm and for chlorophyll *b*, λex = 460 nm and λem = 600−800 nm. Using the Panorama Fluorescence 3.1 software (Lab Cognition, Cologne, Germany), the relative content of chlorophylls *a* and *b* was expressed in conventional units of chlorophyll fluorescence intensity [82].

### 4.6. Determination of the Lipid Peroxidation Level in Tea Callus Cultures

The level of lipid peroxidation in tea callus tissues was determined with the content of malondialdehyde (MDA), using the reaction with thiobarbituric acid (TBA) [83]. For this purpose, a callus frozen in liquid nitrogen (200 mg FW each) was homogenized in 5 mL of 0.1 M tris-HCl buffer (pH 7.5) containing 0.35 M NaCl. One mL of a 0.5% solution of TBA in a 20% aqueous solution of trichloroacetic acid was added to the resulting homogenate. The reaction mixture was incubated in a boiling water bath for 30 min, and the optical density of the solution was measured at 532 nm (spectrophotometer Specord M40, Carl Zeiss, Jena, Germany). To calculate the MDA content (µmol·g^−1^ DW), a molar extinction coefficient of 156 mmol^−1^·cm^−1^ was used [84].

### 4.7. Extraction of Phenolic Compounds from Tea Callus Cultures

PC were extracted from frozen-in-liquid nitrogen calli (50 mg FW each). The extraction was carried out using 96% ethanol (1.5 mL) and a temperature of 45 °C (Gnom thermostat, Moscow, Russia) for 40 min [85]. The homogenate was centrifuged (12,000× *g*, 5 min), and the supernatant was used for the spectrophotometric determination (spectrophotometer Specord M40, Carl Zeiss, Jena, Germany) of the various PC class contents.

### 4.8. Determination of the Total Phenolic Content in Tea Callus Cultures

The determination of the total PC content in ethanol extracts of callus tissues was carried out using the Folin–Ciocalteu reagent (Panreac, Barcelona, Spain, E.U.) according to our modified method [86]. During the determinations, the reaction mixture contained 0.075 mL of callus tissues’ ethanol extract, 0.075 mL of Folin–Ciocolteu reagent, 0.15 mL of 20% Na_2_CO_3_ (*w*/*v*), and 1.20 mL of distilled water. The mixture was stirred and incubated for 45 min in the dark at room temperature. The absorption of the product formed in the reaction (tungsten blue or heteropolyblue) was measured on a spectrophotometer (Specord M40, Carl Zeiss, Jena, Germany) at 725 nm [87]. The results were expressed as mg of gallic acid equivalents (GAE) per g of DW (mg GAE·g^−1^ DW) using a four-point calibration curve of the gallic acid standard (Serva, Heidelberg, Germany).

### 4.9. Determination of the Phenylpropanoid Contents in Tea Callus Cultures

The phenylpropanoid content was evaluated using direct spectrophotometry of callus tissues’ ethanol extracts at 330 nm (spectrophotometer Specord M40, Carl Zeiss, Jena, Germany) due to the characteristic absorption of these substances in the UV region of the spectrum [88]. As a control, we used 96% ethanol. The results were expressed in mg of caffeic acid equivalents (CAE) per g of DW (mg CAE·g^−1^ DW) using a four-point calibration curve of the caffeic acid standard (Serva, Heidelberg, Germany).

### 4.10. Determination of the Flavanol Contents in Tea Callus Cultures

The flavanol content was determined with a vanillin reagent, which is 1% vanillin (Merck, Darmstadt, Germany) in 70% sulfuric acid [89,90]. During the determinations, the reaction mixture contained 0.25 mL of ethanol extract and 1.25 mL of vanillin reagent. The absorption of red adducts formed in this reaction was measured on a spectrophotometer (Specord M40, Carl Zeiss, Jena, Germany) at 500 nm. The results were expressed in mg of epicatechin equivalents (ECE) per g of DW (mg ECE·g^−1^ DW) using a four-point calibration curve of the (−)-epicatechin standard (Serva, Heidelberg, Germany).

### 4.11. Determination of Individual Phenolic Compounds Using the HPLC Method in Tea Callus Cultures

The catechin composition of tea callus cultures was analyzed using the high-performance liquid chromatography (HPLC) method according to the methodology presented previously [91]. Briefly, freeze-dried callus tissues (35–50 mg) were extracted with 80% aqueous methanol, then the supernatant fraction was separated and used for analysis with HPLC with gradient elution using Agilent 1100 series equipment (Agilent, Waldbronn, Germany). A chromatograph was equipped with a flow vacuum degasser, a 4-channel low-pressure gradient pump, an automatic injector, a column thermostat, and a diode array detector [92]. The standards and compounds of callus extracts were separated on a ZORBAX SB-C18 (2.1 × 150 mm) column filled with octadecylsilyl sorbent (grain size 3.5 microns) at 40 °C. The composition of the eluent is as follows: solution A—0.1% aqueous solution of H_3_PO_4_; solution B—90% acetonitrile. The elution gradient is presented in Table S2. At a flow rate of 0.3 mL min−1, the injection amount was 1 μL. The parameters of the removal of the spectrum for catechins were 273 nm. The compounds were identified by comparing their spectral characteristics and retention time with similar characteristics of the standards. The content was calculated using calibration graphs of the peak area dependence on the substance concentration.

### 4.12. Statistical Analysis

All determinations were carried out in three biological and three analytical replicates. The obtained data were statistically processed using Microsoft Excel 2010 14.0 (Redmond, WA, USA) and SigmaPlot 12.2 (Technology Networks, Sudbury, UK) software. The figures show the arithmetic means ± standard deviations (±SDs). The data statistical analyses were performed using the two-way analysis of variance (ANOVA). Mean separation was performed using the normality test (Shapiro–Wilk) and all pairwise multiple comparison procedures (Tukey test). The significant differences at *p* < 0.05 are denoted by different Latin letters, as follows: uppercase letters indicate significant differences between different light intensities in alphabetic order from highest to lowest, and lowercase letters indicate significant differences between the durations of light exposure in alphabetical order from highest to lowest.

## 5. Conclusions

Light is the most important abiotic factor affecting the growth, development, and metabolism of plant cells. It plays an important regulatory role in relation to the biosynthesis of various metabolites and the transition of cells to photomorphogenesis. One cannot exclude its stressful effect on cellular metabolism, which depends on the intensity and duration of light exposure, as well as on the functional activity of the plant system. A change in these parameters initiates a response of plant cells at the level of the antioxidant system, including the formation of low-molecular weight antioxidants, which include phenolic compounds. Despite comprehensive research in this area, some aspects still remain poorly understood, in particular, the effect of primary light exposure of different intensities and durations on the accumulation and balance of various classes of phenolic compounds.

The tea plant (*Camellia sinensis* L.), as well as in vitro cultures obtained from it, are unique objects due to their specialized metabolism aimed at the biosynthesis of phenolic compounds. The most common and significant representatives of the tea plant’s phenolome are flavonols, including catechins, which, along with antioxidant and P-vitamin capillary-strengthening activity, have a number of other valuable beneficial physiological properties.

According to our data, upon primary exposure to light of varying intensities (50, 75, and 100 µmol·m^−2^·s^−1^), changes occurred in the morphology of tea callus cultures without pronounced differences in their growth characteristics and water content as an important parameter of the physiological state of cells. The content of malondialdehyde, as an indicator of cell stress response, in all variants was high and almost equal at the beginning of light exposure and subsequently decreased. During primary light exposure, the accumulation of the total content of phenolic compounds and flavanols at the initial stages of tea calli growth was lower relative to the final stages, in which their level was maximum, which was more pronounced at high light intensity (100 µmol·m^−2^·s^−1^). A different trend is typical for phenylpropanoids (biogenetically early compounds of phenolic metabolism); their greatest accumulation was noted on the 20–30th day of crop growth at lower light intensities (50 and 75 µmol·m^−2^·s^−1^). Based on the data obtained, we can conclude that in tea callus cultures, when they were first exposed to light, the accumulation of phenylpropanoids increased, and only with prolonged exposure did the flavonols increase, and this effect depended on the light’s intensity.

It is important to emphasize that, according to high-performance chromatography data on tea callus cultures, as well as in an intact plant, the catechin complex was represented by both simple (C, EC, EGC) and gallated (GCG, ECG, EGCG) forms, among which the dominant one was EC. The greatest accumulation of catechins was noted at the final stages of growth of tea callus cultures, especially at a low intensity (50 µmol m^−2^·s^−1^).

In general, the obtained results indicate a diverse response of tea callus cultures to different intensities and durations of light exposure. The data we have demonstrated on the absence of a pronounced stress response to light exposure is of interest from the point of view of subsequent studies related to the study of the response of the antioxidant system, represented by both high- and low-molecular weight compounds, including highly effective phenolic antioxidants. Of great importance in both fundamental and applied aspects is the assumption that exposure to different light intensities can be successfully used to further study the biogenesis and expression of the secondary metabolism genes of the tea plant, as well as the modulation of biosynthetic processes of the secondary metabolism and increasing the biological value of plant products.

## Figures and Tables

**Table 1:** Water content in tea callus cultures grown under different intensities of light exposure * during the cultivation cycle (40 days).

Days of Cultivation	Water Content, %		
	R1	R2	R3
1	91.74 ± 0.81 ^Aa^	91.74 ± 0.81 ^Aa^	91.74 ± 0.81 ^Aa^
10	89.77 ± 0.21 ^Ab^	89.08 ± 0.22 ^Bbc^	89.34 ± 0.21 ^Bb^
20	89.32 ± 0.18 ^Ac^	89.60 ± 0.37 ^Ab^	87.43 ± 0.19 ^Bc^
30	89.65 ± 0.30 ^Abc^	86.54 ± 2.45 ^Bc^	87.24 ± 1.65 ^Bc^
40	88.37 ± 0.18 ^Cd^	90.84 ± 0.74 ^Aa^	89.55 ± 0.10 ^Bb^

* Light intensity variants: R1—50 µmol·m^−2^·s^−1^; R2—75 µmol·m^−2^·s^−1^; R3—100 µmol·m^−2^·s^−1^. Results are expressed as means ± SDs, *n* = 3. The significant differences at *p* < 0.05 are denoted by different Latin letters, as follows: uppercase letters indicate significant differences between different light intensities in alphabetic order from highest to lowest, and lowercase letters indicate significant differences between duration of light exposure in alphabetic order from highest to lowest. Pairwise multiple comparisons were carried out using Tukey’s range test.

**Table 2:** The chlorophyll *a* and chlorophyll *b* fluorescence intensity (FI) in tea callus cultures grown under different intensities of light exposure * during the cultivation cycle (40 days).

Variants	Days of Cultivation	Chlorophyll FI, c.u.		
		*a*	*b*	*a* + *b*
R1	10	24.50 ± 4.71 ^Ad^	10.20 ± 1.57 ^Ad^	34.70 ± 6.28 ^Ad^
	20	78.56 ± 5.81 ^Bc^	28.82 ± 2.44 ^Bc^	107.38 ± 8.25 ^Bc^
	30	333.86 ± 57.97 ^ABb^	107.22 ± 39.36 ^ABb^	441.08 ± 97.33 ^ABb^
	40	542.14 ± 22.16 ^Ba^	227.71 ± 9.87 ^Ba^	769.85 ± 32.04 ^Ba^
R2	10	11.97 ± 1.71 ^Bd^	5.86 ± 0.56 ^Bd^	17.83 ± 2.26 ^Bd^
	20	47.07 ± 5.86 ^Cc^	18.98 ± 2.28 ^Bc^	66.05 ± 8.14 ^Cc^
	30	272.93 ± 15.42 ^Bb^	94.24 ± 5.29 ^Bb^	367.17 ± 20.71 ^Bb^
	40	572.45 ± 41.37 ^Ba^	210.63 ± 10.81 ^Ba^	783.08 ± 52.18 ^Ba^
R3	10	6.38 ± 1.07 ^Cd^	4.09 ± 0.58 ^Cc^	10.47 ± 1.66 ^Cd^
	20	279.36 ± 19.71 ^Ac^	93.75 ± 7.27 ^Ab^	373.11 ± 26.98 ^Ac^
	30	339.48 ± 14.23 ^Ab^	111.77 ± 10.85 ^Ab^	451.25 ± 25.08 ^Ab^
	40	788.57 ± 77.90 ^Aa^	271.65 ± 27.87 ^Aa^	1060.22 ± 105.76 ^Aa^

* Light intensity variants: R1—50 µmol·m^−2^·s^−1^; R2—75 µmol·m^−2^·s^−1^; R3—100 µmol·m^−2^·s^−1^. Results are expressed as means ± SDs, *n* = 3. The significant differences at *p* < 0.05 are denoted by different Latin letters, as follows: uppercase letters indicate significant differences between different light intensities in alphabetic order from highest to lowest, and lowercase letters indicate significant differences between duration of light exposure in alphabetic order from highest to lowest. Pairwise multiple comparisons were carried out using Tukey’s range test.

**Table 3:** The individual component contents of the catechin complex in tea callus cultures grown under different intensities of light exposure* during the cultivation cycle (40 days).

		Content of Catechins, mg·g^−1^ DW					
Variants	Days of Cultivation	EGC	C	EC	EGCG	GCG	ECG
R1	1	0.17 ± 0.03 ^Ab^	0.59 ± 0.01 ^Ab^	1.93 ± 0.04 ^Ab^	0.09 ± 0.01 ^Aa^	0.82 ± 0.02 ^Ab^	0.51±0.03 ^Ab^
	10	0.13 ± 0.01 ^Ab^	0.40 ± 0.01 ^Bd^	1.39 ± 0.01 ^Be^	0.09 ± 0.01 ^Ba^	0.66 ± 0.01 ^Ac^	0.35±0.01 ^Ac^
	20	0.08 ± 0.01 ^Ac^	0.33 ± 0.01 ^Be^	1.62 ± 0.01 ^Bc^	0.07 ± 0.01 ^Aa^	0.39 ± 0.06 ^Bd^	0.27±0.06 ^Ac^
	30	0.13 ± 0.01 ^Bb^	0.56 ± 0.01 ^Cc^	1.45 ± 0.01 ^Cd^	0.07 ± 0.01 ^Aa^	0.44 ± 0.05 ^Cd^	0.36±0.09 ^Bc^
	40	0.30 ± 0.01 ^Aa^	1.45 ± 0.01 ^Aa^	4.95 ± 0.01 ^Aa^	0.08 ± 0.01 ^Aa^	1.50 ± 0.01 ^Aa^	0.92±0.01 ^Aa^
R2	1	0.17 ± 0.03 ^Ac^	0.59 ± 0.01 ^Ad^	1.93 ± 0.04 ^Ac^	0.09 ± 0.01 ^Aa^	0.82 ± 0.02 ^Ab^	0.51±0.03 ^Ac^
	10	0.10 ± 0.01 ^Bd^	0.41 ± 0.04 ^ABe^	1.20 ± 0.04 ^Cd^	0.06 ± 0.03 ^Bab^	0.43 ± 0.04 ^Bd^	0.31±0.01 ^Ae^
	20	0.10 ± 0.01 ^Ad^	0.66 ± 0.03 ^Ac^	1.85 ± 0.09 ^Ac^	0.06 ± 0.01 ^Ab^	0.71 ± 0.05 ^Ac^	0.34±0.01 ^Ad^
	30	0.28 ± 0.01 ^Aa^	1.01 ± 0.04 ^Aa^	3.45 ± 0.11 ^Aa^	0.05 ± 0.01 ^Ab^	1.13 ± 0.04 ^Aa^	0.77±0.02 ^Aa^
	40	0.23 ± 0.01 ^Bb^	0.77 ± 0.03 ^Cb^	2.61 ± 0.04 ^Cb^	0.03 ± 0.02 ^Ba^	0.82 ± 0.05 ^Cb^	0.60±0.01 ^Bb^
R3	1	0.17 ± 0.03 ^Ac^	0.59 ± 0.01 ^Ac^	1.93 ± 0.04 ^Ab^	0.09 ± 0.01 ^Ab^	0.82 ± 0.02 ^Ab^	0.51±0.03 ^Aa^
	10	0.11 ± 0.01 ^ABc^	0.43 ± 0.01 ^Ad^	1.60 ± 0.01 ^Ac^	0.13 ± 0.01 ^Aa^	0.64 ± 0.01 ^Ac^	0.31±0.01 ^Ab^
	20	0.08 ± 0.01 ^Ad^	0.22 ± 0.01 ^Ce^	0.75 ± 0.01 ^Cd^	0.06 ± 0.01 ^Ac^	0.23 ± 0.02 ^Ce^	0.20±0.04 ^Bc^
	30	0.15 ± 0.02 ^Bb^	0.87 ± 0.03 ^Bb^	1.69 ± 0.08 ^Bc^	0.06 ± 0.01 ^Ac^	0.55 ± 0.03 ^Bd^	0.31±0.01 ^Bb^
	40	0.22 ± 0.01 ^Ba^	1.25 ± 0.01 ^Ba^	3.23 ± 0.01 ^Ba^	0.03 ± 0.01 ^Bd^	0.96 ± 0.01 ^Ba^	0.21±0.01 ^Cc^
Retention time, min		13.23	13.65	15.33	15.54	15.67	17.36

* Light intensity variants: R1—50 µmol·m^−2^·s^−1^; R2—75 µmol·m^−2^·s^−1^; R3—100 µmol·m^−2^·s^−1^. EGC—epigallocatechin; C—(+)-catechin; EC—(−)-epicatechin; EGCG—epigallocatechin gallate; GCG—gallocatechin gallate; ECG—epicatechin gallate. Results are expressed as means ± SDs, *n* = 3. The significant differences at *p* < 0.05 are denoted by different Latin letters, as follows: uppercase letters indicate significant differences between different light intensities in alphabetic order from highest to lowest, and lowercase letters indicate significant differences between duration of light exposure in alphabetic order from highest to lowest. Pairwise multiple comparisons were carried out using Tukey’s range test.

## Data Availability

The data presented in this study are available on request from the corresponding author. Data are contained within the article or Supplementary Materials.

## Supplementary Materials

The following supporting information can be downloaded at https://www.mdpi.com/article/10.3390/ijms251910420/s1.
