# Supplementary material for: Influence of Primary Light Exposure on the Morphophysiological Characteristics and Phenolic Compounds Accumulation of a Tea Callus Culture (Camellia sinensis L.)
Source: Int J Mol Sci. 2024 Sep 27;25(19):10420. doi: 10.3390/ijms251910420 (PMC11477156; doi:10.3390/ijms251910420)
Supplement: Supplementary file 1 [file ijms-25-10420-s001.zip › ijms-3105045-supplementary.pdf]

R1 R2 R3

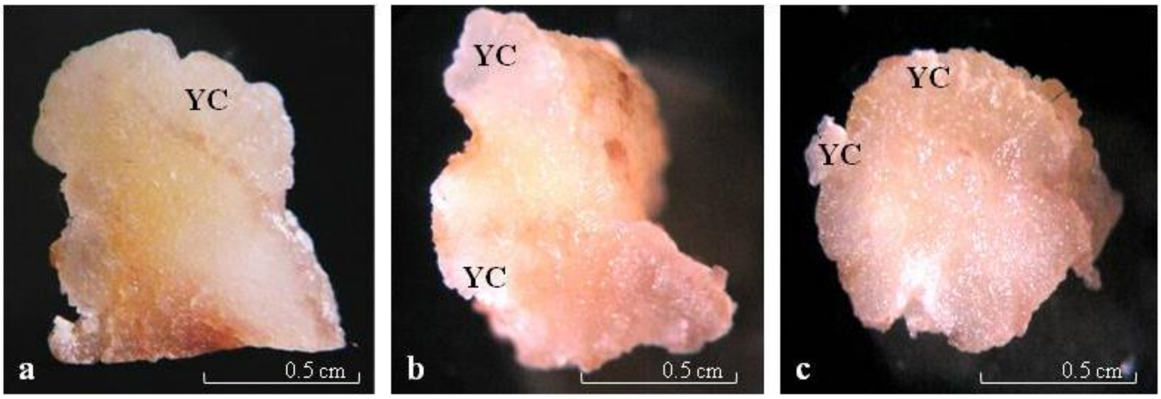


**Figure S1.** Lateral sections of tea callus calluses grown under different intensity of light exposure: R1 – 50 µmol·m$^{-2}$·s$^{-1}$ (a); R2 – 75 µmol·m$^{-2}$·s$^{-1}$ (b); R3 – 100 µmol·m$^{-2}$·s$^{-1}$ (c). Duration of light exposure – 10 days. Symbol: YC -young cells.

**Figure S2.** HPLC chromatograms of the catechin complex individual components in tea callus cultures grown under different intensity of light exposure (10 and 40 days): R1 – 50 µmol·m$^{-2}$·s$^{-1}$; R2 – 75 µmol·m$^{-2}$·s$^{-1}$; R3 – 100 µmol·m$^{-2}$·s$^{-1}$. EGC – epigallocatechin; GCG – gallocatechin gallate; ECG – epicatechin gallate; EGCG – epigallocatechin gallate. a – 1 day; b – R1, 10 days; c – R1, 40 days; d – R2, 10 days; e –R2, 40 days; f – R3, 10 days; g – R3, 40 days.

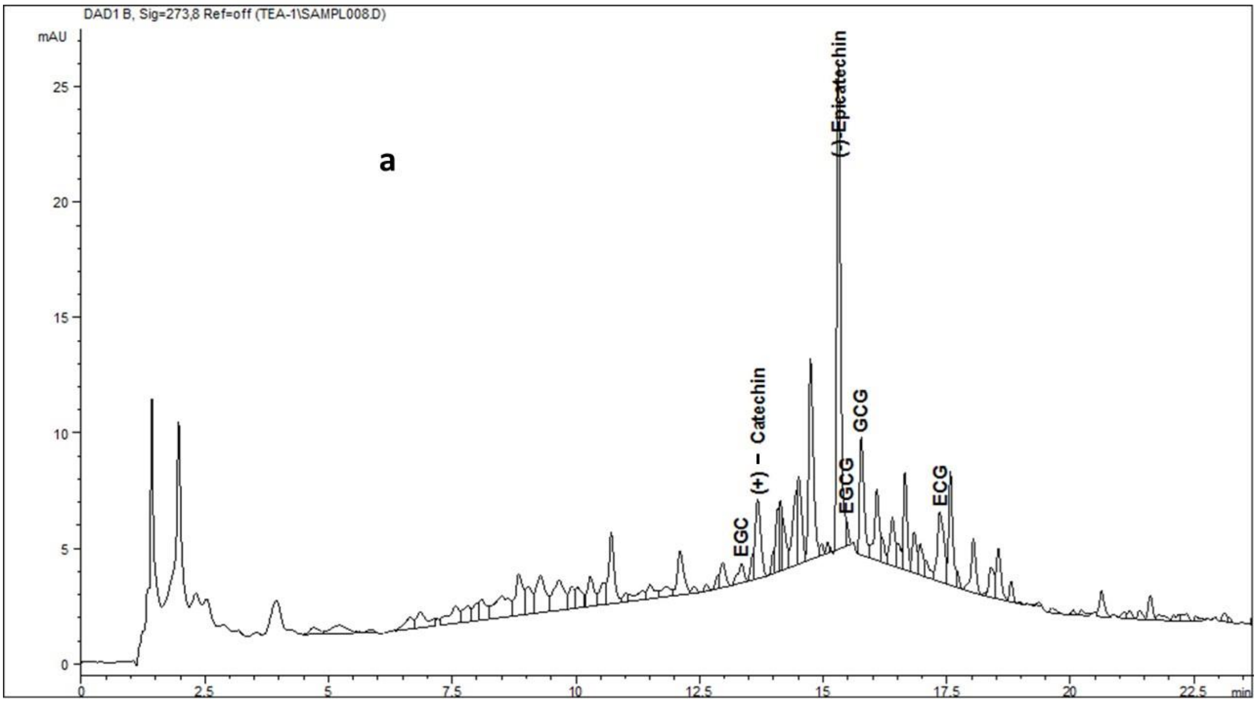


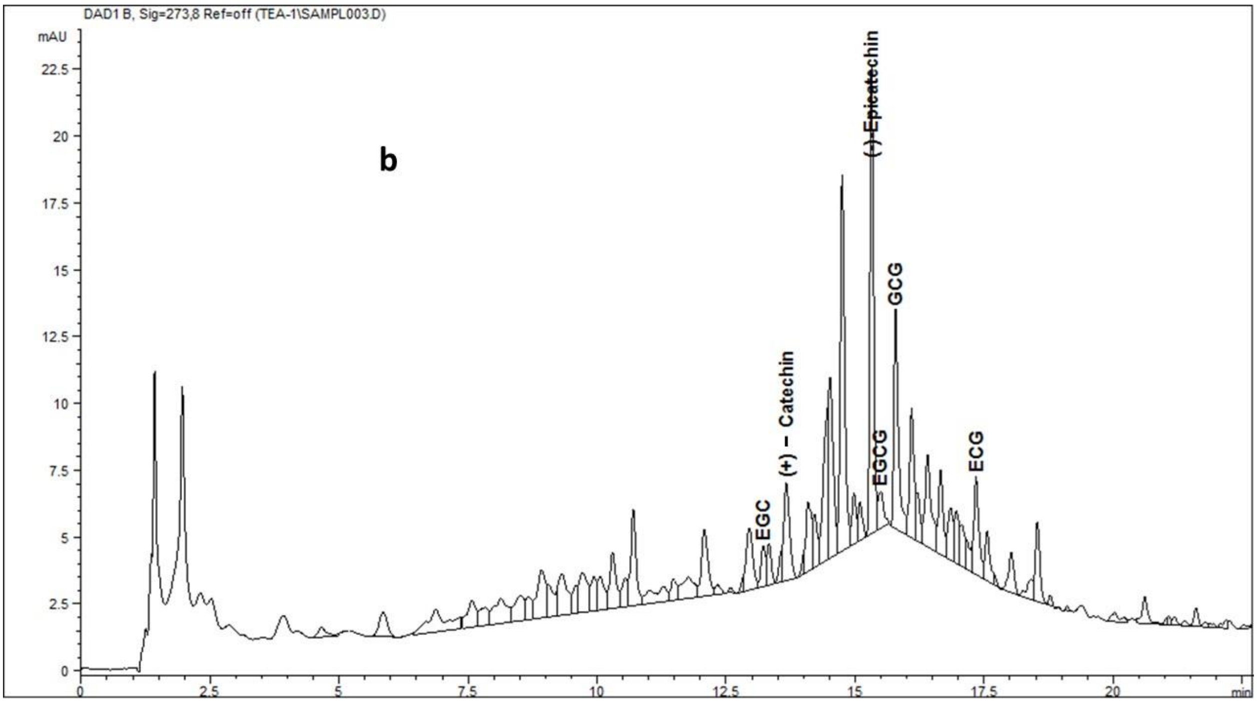

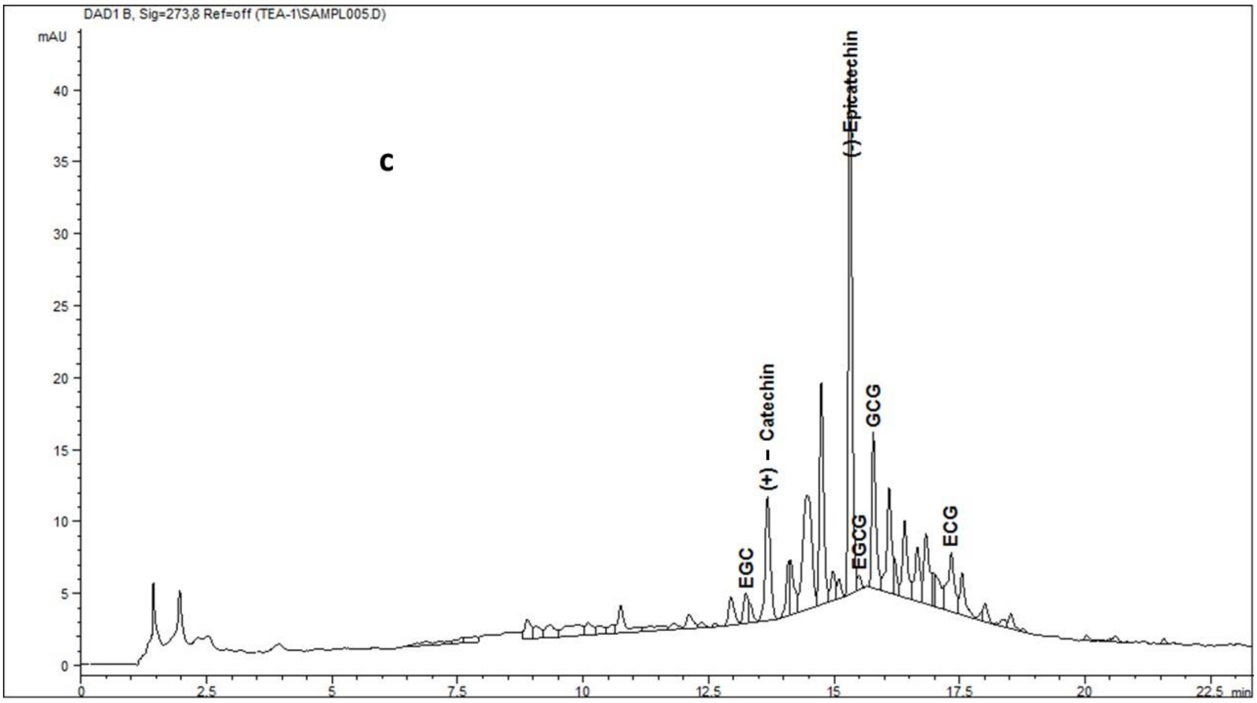

DAD1 B, Sig=273,8 Ref=off (TEA-1\SAMPL005.D)
mAU
c
(-)-Epicatechin
(+) – Catechin
GCG
EGC
EGCG
ECG
min


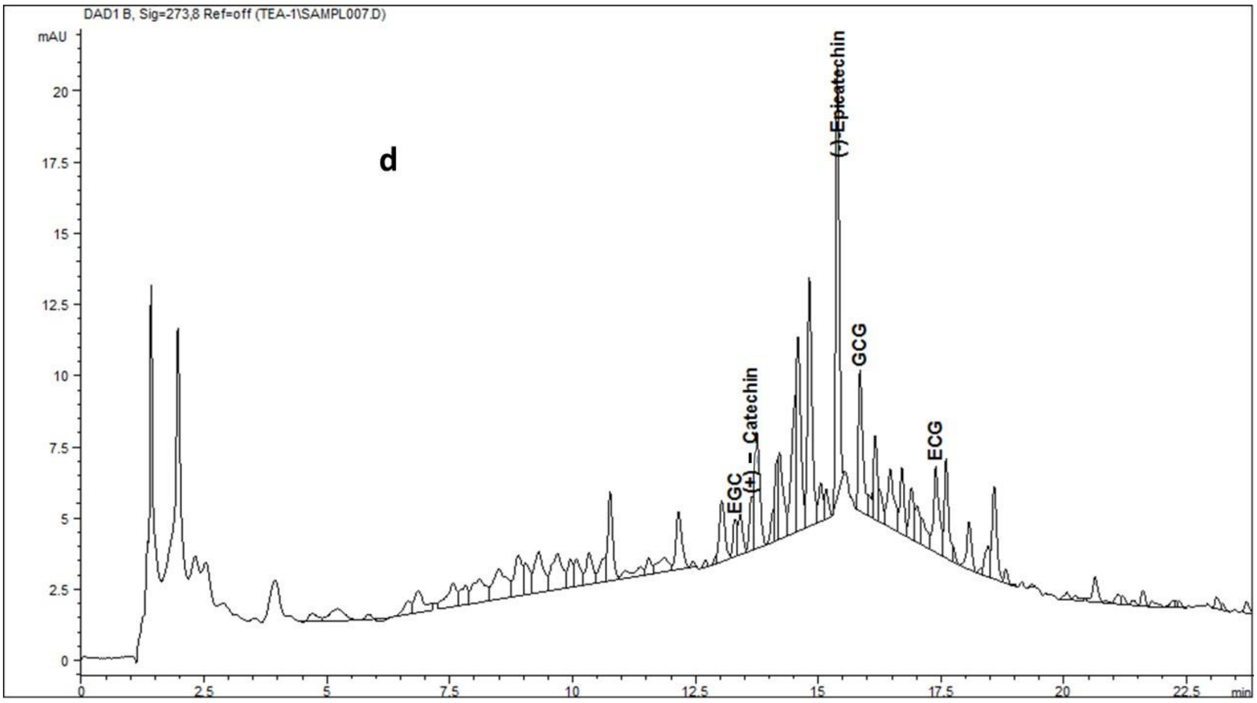

DAD1 B, Sig=273,8 Ref=off (TEA-1\SAMPL007.D)
mAU
d
(-)-Epicatechin
GCG
(+) – Catechin
EGC
ECG
min

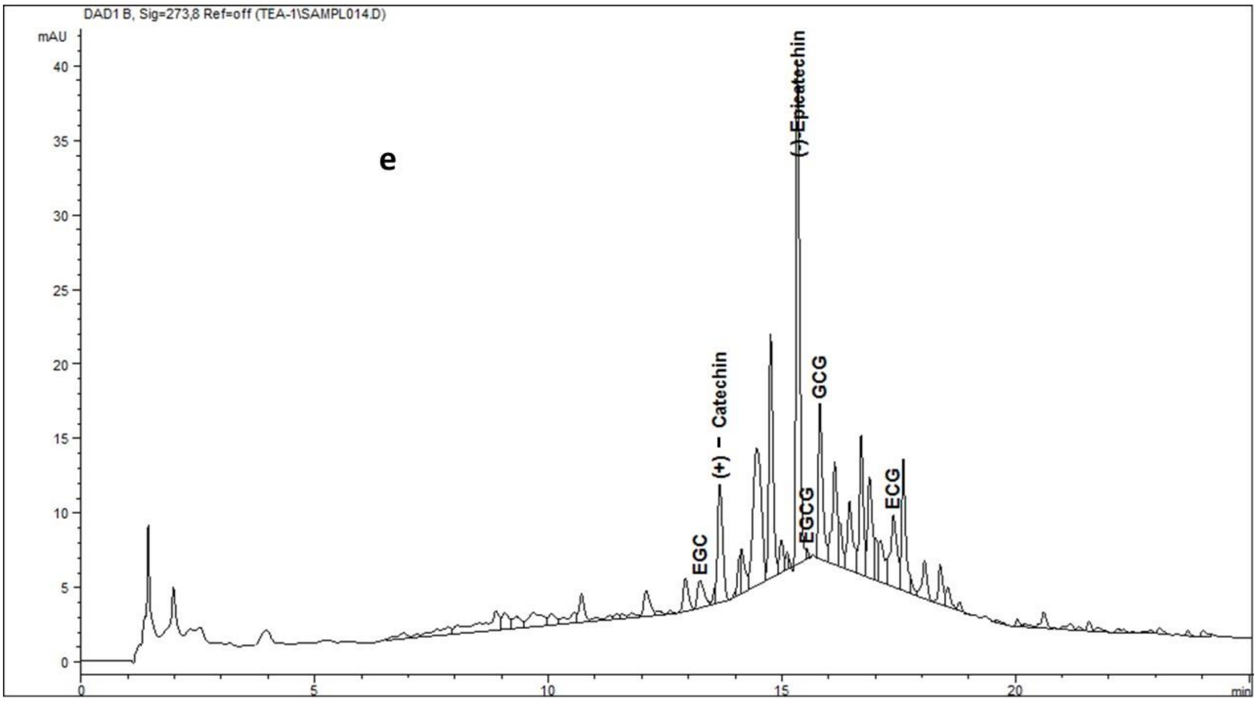
DAD1 B, Sig=273,8 Ref=off (TEA-1\SAMPL014.D)
mAU
e
(-)-Epicatechin
(+) – Catechin
GCG
EGCG
ECG
EGC
min

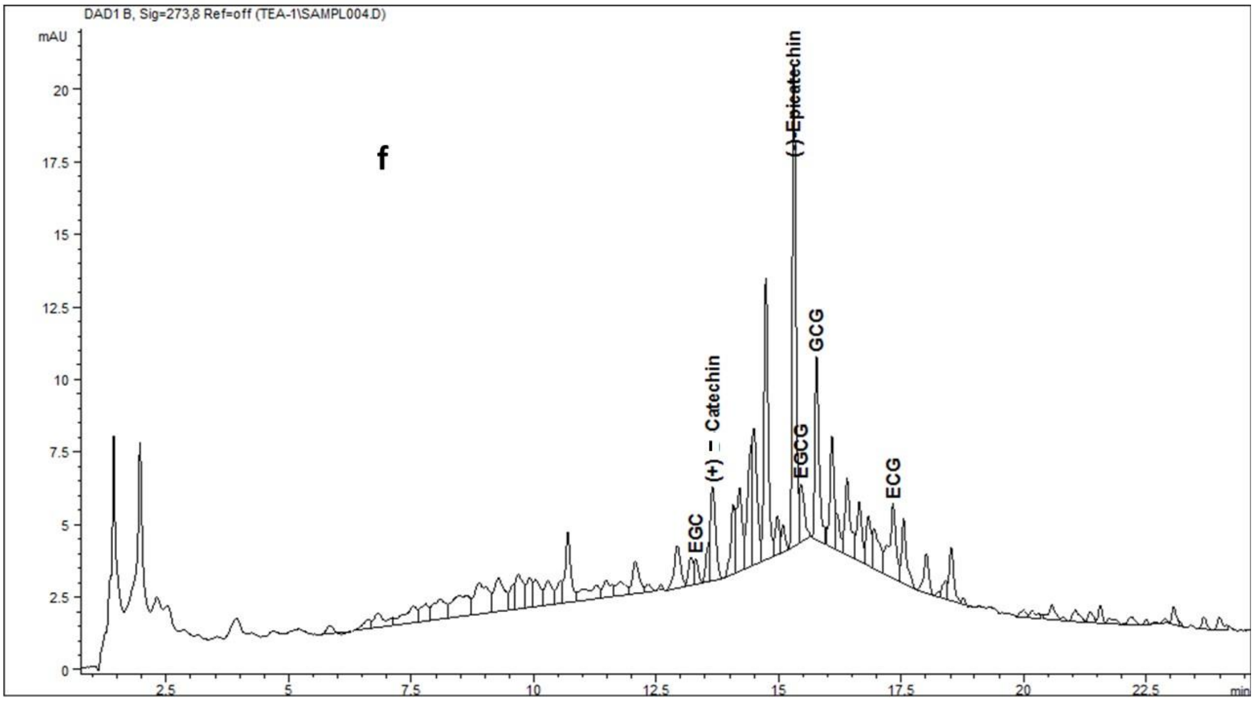
DAD1 B, Sig=273,8 Ref=off (TEA-1\SAMPL004.D)
mAU
f
(-)-Epicatechin
GCG
(+) – Catechin
EGCG
ECG
EGC
min

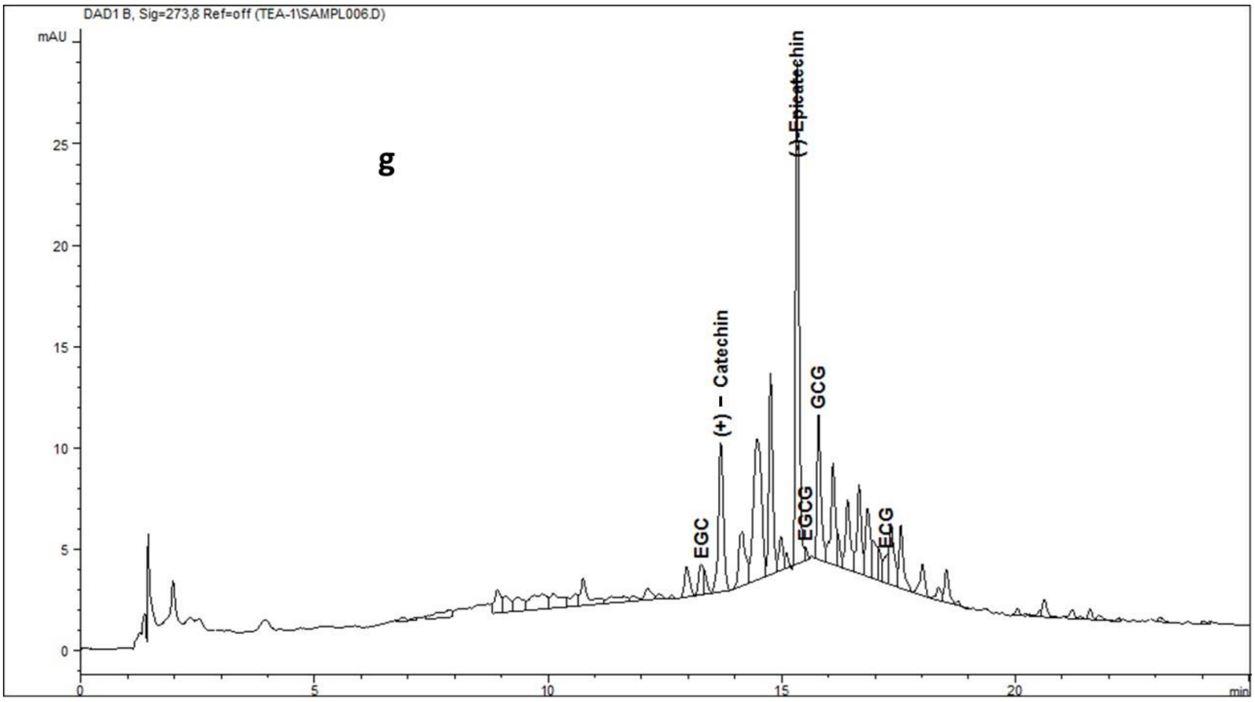
DAD1 B, Sig=273,8 Ref=off (TEA-1\SAMPL006.D)
mAU
g
(-)-Epicatechin
(+) – Catechin
GCG
EGC
EGCG
ECG
min

**Table S1.** Two-way ANOVA showing the effect of the light intensity and time of cultivation and their interaction on water, phenolic compounds, malondialdehide, chlorophyll *a* and *b* and catechin complex components contents in tea callus cultures.

| Variable and source of variation | df | F | P | Variable and source of variation | df | F | P |
|---|---|---|---|---|---|---|---|
| *Water content* | | | | *(-)-Epicatechin content* | | | |
| *Light intensity* | 3 | 1.158 | 0.341 | *Light intensity* | 3 | 464.679 | <**0.001** |
| *Time of cultivation* | 3 | 1.370 | 0.270 | *Time of cultivation* | 3 | 3927.226 | <**0.001** |
| *Light intensity × time of cultivation* | 9 | 1.180 | 0.341 | *Light intensity × time of cultivation* | 9 | 1418,402 | <**0.001** |
| *Total phenolic content* | | | | *(+)-Catechin content* | | | |
| *Light intensity* | 3 | 2.511 | 0.068 | *Light intensity* | 3 | 125.014 | <**0.001** |
| *Time of cultivation* | 3 | 22.770 | <**0.001** | *Time of cultivation* | 3 | 3087.163 | <**0.001** |
| *Light intensity × time of cultivation* | 9 | 5.496 | <**0.001** | *Light intensity × time of cultivation* | 9 | 865.601 | <**0.001** |
| *Phenylpropanoids content* | | | | *Epigallocatechin content* | | | |
| *Light intensity* | 3 | 49.412 | <**0.001** | *Light intensity* | 3 | 7.661 | <**0.001** |
| *Time of cultivation* | 3 | 166.305 | <**0.001** | *Time of cultivation* | 3 | 102.661 | <**0.001** |
| *Light intensity × time of cultivation* | 9 | 25.641 | <**0.001** | *Light intensity × time of cultivation* | 9 | 26.410 | <**0.001** |
| *Flavanols content* | | | | *Gallocatechin gallate content* | | | |
| *Light intensity* | 3 | 4.654 | 0.006 | *Light intensity* | 3 | 124.461 | <**0.001** |
| *Time of cultivation* | 3 | 14.137 | <**0.001** | *Time of cultivation* | 3 | 661.578 | <**0.001** |
| *Light intensity × time of cultivation* | 9 | 4.328 | <**0.001** | *Light intensity × time of cultivation* | 9 | 325.300 | <**0.001** |
| *Malondialdehide content* | | | | *Epicatechin gallate content* | | | |
| *Light intensity* | 3 | 0.010 | 0.090 | *Light intensity* | 3 | 184.420 | <**0.001** |
| *Time of cultivation* | 3 | 2.406 | 0.092 | *Time of cultivation* | 3 | 143.176 | <**0.001** |
| *Light intensity × time of cultivation* | 9 | 1.795 | 0.143 | *Light intensity × time of cultivation* | 9 | 103.621 | <**0.001** |
| *Chlorophyll a FI* | | | | *Epigallocatechin gallate* | | | |
| *Light intensity* | 3 | 22.830 | <**0.001** | *Light intensity* | 3 | 35.441 | <**0.001** |
| *Time of cultivation* | 3 | 303.518 | <**0.001** | *Time of cultivation* | 3 | 27.988 | <**0.001** |
| *Light intensity × time of cultivation* | 9 | 7.118 | <**0.001** | *Light intensity × time of cultivation* | 9 | 13.459 | <**0.001** |
| *Chlorophyll b FI* | | | | | | | |
| *Light intensity* | 3 | 13.410 | <**0.001** | | | | |
| *Time of cultivation* | 3 | 302.171 | <**0.001** | | | | |
| *Light intensity × time of cultivation* | 9 | 3.950 | **0.003** | | | | |

**Figure S3.** The spectra of the Philips TL–D 58 W/33-640 fluorescent lamps (Poland) were used in the experiment*

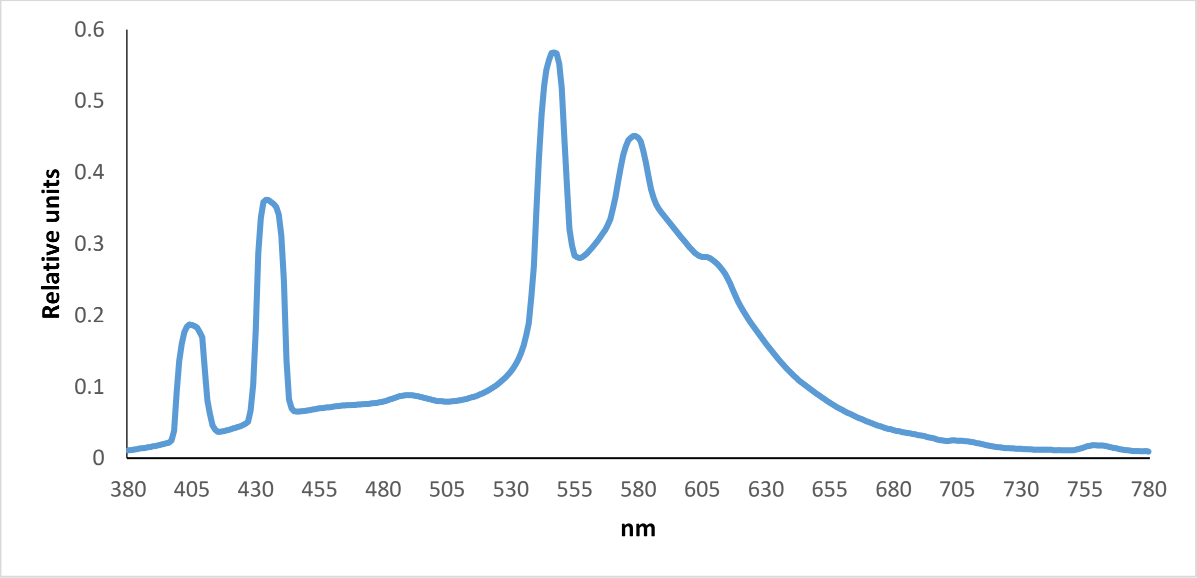


*The spectra of the lamps was recorded using LI-180 Spectrometer (LI-COR, Lincoln, Nebraska, USA).

**Table S2.** The HPLC gradient mode.

| Time, min | A*, % | B*, % |
|---|---|---|
| 0,0 | 100 | 0 |
| 8,0 | 90 | 10 |
| 25,0 | 40 | 60 |
| 25,1 | 0 | 100 |
| 29,0 | 0 | 100 |
| 29,1 | 100 | 0 |
| 34,00 | 100 | 0 |

*A—0.1% aqueous solution of H3PO4, B—90% acetonitrile
